# Effectiveness of composting as a biosecure disposal method for porcine epidemic diarrhea virus (PEDV)-infected pig carcasses

**DOI:** 10.1186/s40813-017-0068-z

**Published:** 2017-11-28

**Authors:** Sarah Vitosh-Sillman, John Dustin Loy, Bruce Brodersen, Clayton Kelling, Kent Eskridge, Amy Millmier Schmidt

**Affiliations:** 1 School of Veterinary Medicine and Biomedical Sciences, University of Nebraska-Lincoln, Fair Street and East Campus Loop, Lincoln, NE 68583 USA; 2 Department of Statistics, University of Nebraska-Lincoln, Lincoln, NE 68583 USA; 3 Department of Biological Systems Engineering and Department of Animal Science, University of Nebraska-Lincoln, Lincoln, NE 68583 USA

**Keywords:** Porcine epidemic diarrhea virus, Composting, Temperature, Mortality, Carcass disposal

## Abstract

**Background:**

Porcine epidemic diarrhea virus (PEDV) is an enteric disease of swine that has emerged as a worldwide threat to swine herd health and production. Substantial research has been conducted to assess viability of the virus on surfaces of vehicles and equipment, in feed and water, and on production building surfaces, but little is known about the persistence in PEDV-infected carcasses and effective disposal methods thereof. This study was conducted to quantify the persistence of PEDV RNA via quantitative real-time reverse transcription polymerase chain reaction (qRT-PCR) at various time-temperature combinations and in infected piglet carcasses subjected to composting. Although this method does not distinguish between infectious and noninfectious virus, it is a rapid and sensitive test to evaluate materials for evidence of virus genome.

**Results:**

In the first study, PEDV was suspended in cell culture media at 1 × 10^5^ TCID50 per sample (1 mL sample size) and subjected to various time and temperature combinations in triplicate including temperatures of 37, 45, 50, 55, 60, 65, 70 °C and exposure times of 0, 1, 2, 3, 4, 5, 7, and 14 days. At all temperatures, viral RNA copies declined over time, with the decline most marked and rapid at 65 and 70 °C. Detectable RNA did persist throughout the trial in all but the most extreme condition, where two of three samples incubated at 70 °C yielded undetectable viral RNA after 14 days. In the second study, PEDV-infected piglet carcasses were subjected to two cycles of composting lasting 36 and 37 days, respectively, for a total compost time of 73 days. Composting was performed in triplicate windrow sections housed inside biosecure, climate-controlled rooms using insulated bins designed to represent a continuous windrow compost pile. Temperatures reached 35–57 °C for 26 days of cycle 1 and 35–45 °C for 3 days of cycle 2. Samples consisting of carbon material with or without decomposed tissue as available per sample site collected at ten locations throughout the cross-section of each windrow section following the primary and secondary compost cycles yielded no detectable viral RNA.

**Conclusions:**

Composting appears to be an effective disposal method for PEDV-infected piglet carcasses under the conditions examined. The combination of time and high temperature of the compost cycle effectively degraded viral RNA in cell culture media that should provide optimum stability. Complex compost material matrices collected from windrow sections yielded undetectable PEDV RNA by qRT-PCR after one 36-day compost cycle despite incomplete decomposition of soft tissue.

## Background

Porcine epidemic diarrhea virus (PEDV), an RNA virus of the family *Coronaviridae*, genus *Alphacoronavirus*, causes an economically devastating enteric disease of swine [1]. The virus can infect all ages of swine and is characterized by clinical disease of watery diarrhea, vomiting, and dehydration, with high morbidity and mortality – often near 100% – in naïve suckling piglets [2]. In May 2013, PEDV was detected in outbreaks of porcine diarrhea in Iowa, United States, and quickly spread to over half of the states in the U.S. and subsequently to Canada [3, 4]. Prior to this outbreak, cases of PEDV infection had been limited to Europe and Asia [5]. Extensive investigation into how PEDV arrived in the U.S. and quickly disseminated throughout the swine industry has implicated PEDV-contaminated feed or feed ingredients as the potential origin of virus introduction [6–9]. A comprehensive root cause investigation report organized by the United States Department of Agriculture (USDA) identified flexible intermediate bulk containers, commonly used to transport feed ingredients, as the most likely source of PEDV introduction to the country [10]. These findings demonstrate the critical need to understand PEDV persistence in multiple complex matrices, including those in the environment, so that transmission of the disease can be prevented.

With nearly 100% mortality in pre-weaned piglets, the large number of carcasses and volume of infectious material generated during PEDV outbreaks was substantial. Proven, biosecure methods for carcass disposal are sought to control on-farm virus proliferation and limit site-to-site transmission. Major mortality disposal methods available to swine producers in the U.S. include rendering, incineration, burial, land-filling, and composting [11]. Although composting is widely accepted in the U.S. and Canada, it is not legally permitted for swine producers in the European Union, which are primarily limited to the methods of rendering and incineration [12]. Composting requires relatively low input costs, poses little environmental risk when properly designed and managed, and offers greater biosecurity than methods involving transport of infected carcasses beyond the farm boundary. Furthermore, in situations of disease-associated mortalities, composting is capable of inactivating the pathogen of concern when the system is managed to achieve and maintain pile temperature targets. Several studies have demonstrated this concept with carcasses and manure from virus-infected animals. Windrow-composted poultry manure from Newcastle disease virus (NDV)-infected birds was found to be free of detectable virus by virus isolation [13]. Avian influenza (AI) and NDV were inactivated following 7 days of composting at temperatures reaching 50–65 °C [14]. Compost piles containing AI-infected chicken carcasses were determined AI-negative by virus isolation after 10 days [15]. Pseudorabies virus-infected pig carcasses composted at temperatures that reached 27 to 51 °C contained undetectable virus by isolation techniques in samples collected at days 7 and 14, and compost samples collected after 35 days of composting were negative via bioassay conducted on naïve exposed sentinel pigs [16]. For pig carcasses infected with foot-and-mouth disease (FMD) virus, a more environmentally stable non-enveloped virus than those previously discussed, compost pile temperatures reaching 50 °C at 10 days of composting was successful at virus inactivation [17]. Given these demonstrated successes, the USDA published guidance to promote composting as a disposal method for birds infected by highly pathogenic AI in response to outbreaks of this disease in 2016 [18]. Overall, composting has been proven effective for managing important viral pathogens in animal production waste products by multiple testing modalities and may also be a biosecure on-farm disposal method for use by swine producers to control PEDV transmission.

PEDV is an enveloped virus that is demonstrably sensitive to a variety of disinfectants, extremes of pH, and elevated temperature [19, 20]. Therefore, exposing PEDV-infected dead animals to the elevated temperatures during routine composting may be an effective method of virus elimination. The present study was performed to evaluate the persistence of PEDV RNA with qRT-PCR in matrices and temperature conditions representative of composting in order to determine the potential effectiveness of this method for PEDV mortality disposal. The study consisted of two trials: a laboratory phase examining the rate of virus RNA degradation under a controlled application of time and temperature combinations in physiologic media; and a composting phase where PEDV-infected piglet carcasses were incorporated into compost windrow sections and monitored for temperature and virus degradation over two composting cycles.

## Results

### Time-temperature trial qRT-PCR

**Fig. 1:**
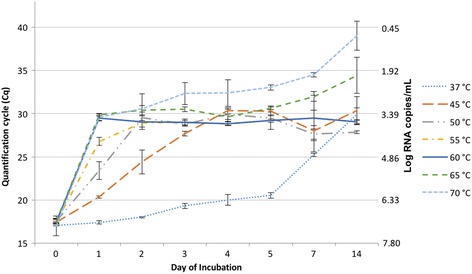
Mean (*n* = 3) quantification cycle and RNA concentration over time by treatment. Error bars represent SD

PEDV RNA was detectable at all time and temperature combinations tested, except for two of three samples on day 14 at 70 °C. Viral RNA reduction kinetics varied greatly among temperature treatments; however, the rate of reduction peaked after the initial days of temperature treatments above 37 °C and then slowed (Table 1). This is reflected by the increase in mean quantification cycle (Cq) of the qRT-PCR over time, which corresponds with a reduction in RNA targets in the sample (Fig. 1). At 37 °C, viral RNA steadily degraded at an average rate of 0.29 log per day throughout the temperature treatment. The amount of viral RNA detected from day 1 to 7 at 37 °C was significantly different from all other temperature treatments on those days. At 45 °C, viral RNA reduction was more rapid from days 0 to 4 (average 1 log reduction per day) with no mean log change thereafter. The 50 °C treatment yielded a mean rate of RNA reduction from days 0 to 2 of 1.87 log of RNA per day, but the remainder of time treatment (2 to 14 days) did not reveal further significant decrease of viral RNA. At 55 °C, RNA decreased by 2.91 log during the first day of treatment, 0.68 log during the second day, and remained generally stable from days 2 through 14 with no further significant decrease in RNA. For 60, 65, and 70 °C treatments, viral RNA reduction was greatest – between 3.72 and 3.9 log – in the first day with no difference (*p* < .05) among the temperatures. From 1 to 14 days, viral RNA decreased a total of 0.28, 1.42, and 2.44 log at 60, 65, and 70 °C, respectively. Despite the similar rapid decrease at day 1, the quantity of viral RNA remaining at day 14 was significantly different among all of these high treatment temperatures.

**Table 1:** Summary of time-temperature trial qRT-PCR assay results as RNA copy equivalents

	qRT-PCR mean log RNA copies/mL (± SD)						
Days	37 °C	45 °C	50 °C	55 °C	60 °C	65 °C	70 °C
0	8.57 (0.02)	8.43 (0.02)	8.40 (0.08)	8.44 (0.03)	8.41 (0.08)	8.41 (0.11)	8.57 (0.36)
1	8.45 (0.05)	7.53 (0.04)	6.58 (0.32)	5.53 (0.15)	4.69 (0.11)	4.58 (0.51)	4.67 (0.12)
2	8.26 (0.03)	6.27 (0.42)	4.67 (0.10)	4.85 (0.25)	4.83 (0.02)	4.41 (0.60)	4.35 (0.12)
3	7.85 (0.09)	5.25 (0.08)	4.91 (0.06)	4.85 (0.13)	5.03 (0.08)	4.37 (0.08)	3.81 (0.40)
4	7.64 (0.19)	4.43 (0.11)	4.56 (0.19)	4.89 (0.07)	5.16 (0.06)	4.65 (0.32)	3.79 (0.48)
5	7.48 (0.11)	4.43 (0.17)	4.69 (0.11)	4.79 (0.31)	4.60 (0.06)	4.34 (0.05)	3.58 (0.09)
7	6.02 (0.06)	5.16 (0.75)	5.27 (0.19)	4.69 (0.82)	5.09 (0.28)	3.91 (0.18)	3.13 (0.07)
14	4.55 (0.22)	4.43 (0.50)	5.21 (0.05)	4.83 (0.05)	4.41 (0.09)	3.16 (0.65)	0.78 (0.13)^a^

^a^2 of 3 samples negative (below detection limit of 8.8 RNA copies per reaction)

### Piglet infection

All piglets exhibited clinical diarrhea by day 3 post-inoculation. On day 5 post-inoculation, which coincided with infection period termination and necropsy, all piglets incorporated into the compost windrow sections displayed a diffusely dilated and thin-walled small intestine with fluidic intestinal content, which was considered consistent with PEDV infection. Microscopically, there was evident atrophic enteritis. All piglets were qRT-PCR positive for PEDV on fecal swab (mean Cq 23.07, range 19.93 to 34.75) and were strongly positive by PEDV immunohistochemistry in the small intestine and mesenteric lymph node.

### Compost pile temperature performance

**Fig. 2:**
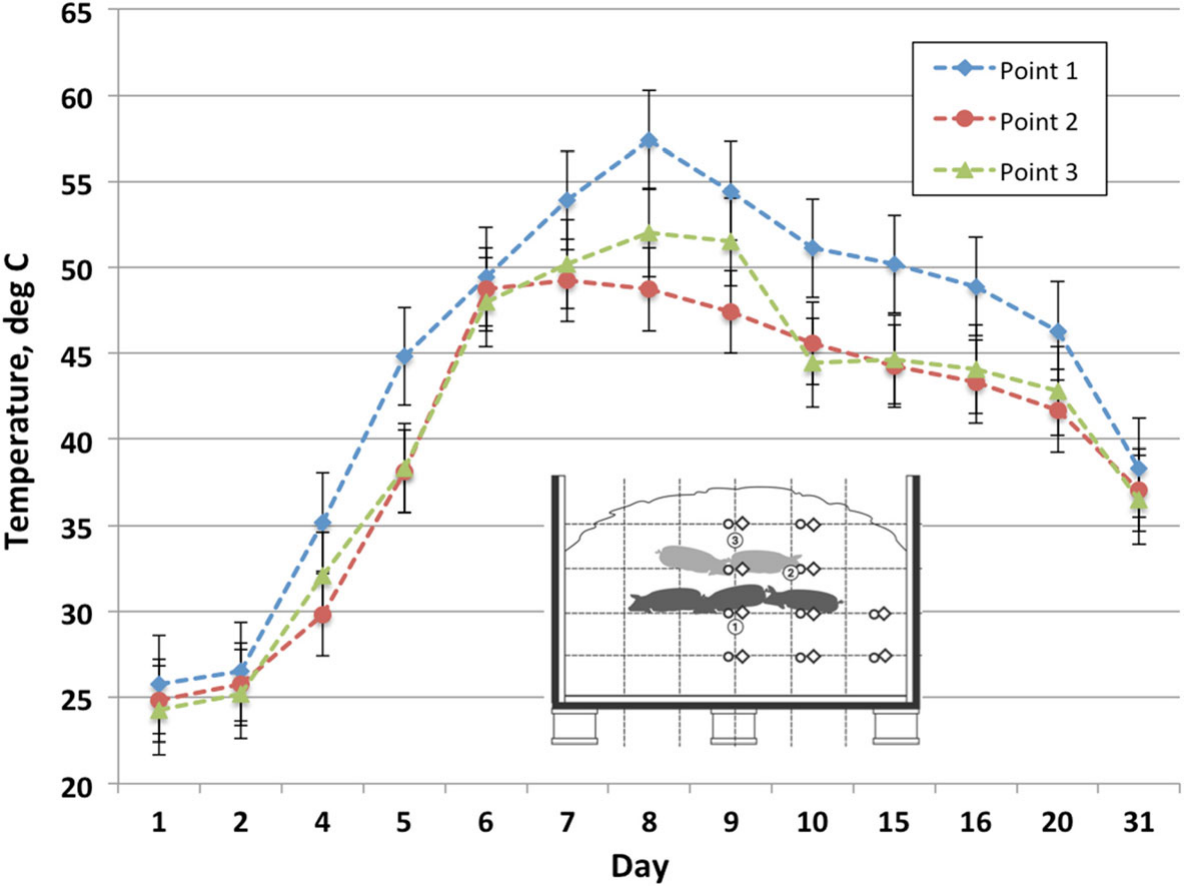
Mean (*n* = 3) manually recorded temperatures of compost windrow sections during compost cycle 1

**Fig. 3:**
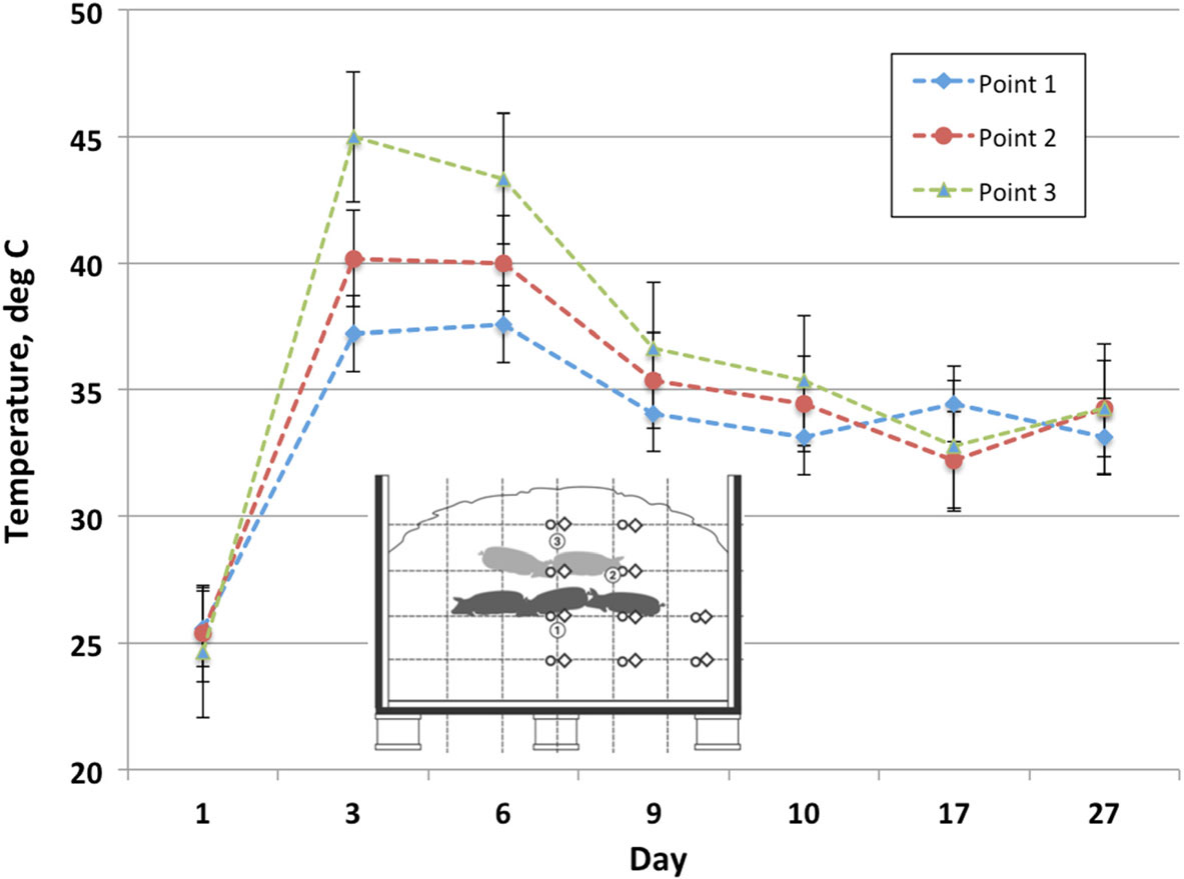
Mean (*n* = 3) manually recorded temperatures of compost windrow sections during compost cycle 2

Despite prior testing of temperature loggers under prolonged high temperature and moisture conditions, the majority of the loggers failed early in the composting process for both compost cycles. The temperature loggers were not monitored continuously, nor were they periodically checked throughout the trial for function, to avoid disturbing their position, so the failure was not realized until the end of the trial. To ensure robust data collection, manual temperature monitoring was also performed and temperatures recorded throughout both compost cycles. Prior to failure during compost cycle 1, temperatures of 45 to 58 °C were recorded at the core of each compost windrow section. Mean manually recorded temperatures of windrow sections for cycles 1 and 2 are illustrated in Figs. 2 and 3, respectively. Manual temperature monitoring throughout the duration of each compost cycle revealed that mean windrow section temperatures of at least 40 °C (104 °F) were sustained for at least 14 d at all points monitored during cycle 1, while a mean temperature of at least 50 °C (122 °F) was sustained for 9 d at point 1 during cycle 1. During cycle 2, a mean temperature of 40 °C (104 °F) was achieved at points 2 and 3 for approximately 3 d, while 50 °C (122 °F) was not attained for any of the monitoring points in the windrow sections.

Using the EPA 503b rule as the criteria for determining successful composting of the carcasses for microbial reductions, benchmarks of 40 °C for 120 h (5 d) and 55 °C for 4 h must be achieved [21]. From the data presented, windrow sections achieved the benchmark of 40 °C for at least 120 h during compost cycle 1, but only the core of the windrow sections achieved 55 °C for greater than 4 h. During compost cycle 2, points 2 and 3 reached a mean temperature of 40 °C for approximately 72 h, which falls short of the EPA 503b rule criteria by 48 h, while no points achieved the benchmark of 55 °C for 4 h.

### Detection of PEDV RNA in mortality composting pile

PEDV RNA was not detected by qRT-PCR in any of the compost samples collected following the first or second compost cycles.

## Discussion

Elimination of PEDV RNA in composted pig carcasses achieved in this study is comparable to others demonstrating genomic RNA degradation in viruses directly subjected to composting, although experimental conditions may vary considerably. AI- and NDV-spiked chicken litter composted at temperatures reaching 50–65 °C were void of detectable viral RNA in 10 days [14]. For FMD virus associated with infected pig carcasses, composting at temperatures reaching 50–70 °C for several days was able to degrade viral RNA below RRT-PCR detection by day 21 [17]. FMD, a non-enveloped virus, is likely more environmentally persistent and resistant to organic solvents than enveloped viruses such as AI, NDV, and PEDV [22]. Therefore, the absence of detectable PEDV RNA in samples from the end of the first compost cycle at day 36 in this study is consistent with previous reports and is a reasonable result considering the variable physical properties of viruses. One problem with making a direct comparison between studies is differences in compost pile temperatures, especially when time and temperature treatment is presumed to be a vital component of pathogen destruction. The compost windrow sections in this study did not consistently achieve published compost temperature benchmarks or comparable maximum temperatures, as a mean temperature of 55 °C for 4 h was only achieved at the core of the windrow sections during cycle 1. While the temperature profile of compost piles does affect the rate of decomposition and pathogen inactivation, virus degradation in compost material has also been shown to partly rely on factors other than temperature, such as microbial activity. AI and NDV in sealed vials subjected to composting were detected by RRT-PCR at the end of testing on day 21, whereas the viruses spiked into chicken manure, used litter, feed, and a homogenate of virus-infected embryonated chicken eggs contained in mesh bags and composted were not detectable by day 10 [14]. In the same experiment, virus-inoculated embryonated chicken eggs that remained intact during composting contained detectable viral RNA, but RNA had degraded beyond detection in those that were crushed. Similarly, with virus survival, another study where NDV and avian encephalomyelitis virus (AEV) were composted in sealed vials or permeable cassettes demonstrated markedly reduced virus survival within the cassettes [23]. NDV in cassettes survived no more than 7 days, while the virus in vials survived up to 28 days. Likewise, AEV in cassettes was inactivated in about 7 days, while the vials contained infectious virus for at least 49 days. Therefore, the exposure of viruses to the complex microbial environment and decomposition by-products of compost is associated with accelerated pathogen destruction beyond the effects of temperature. Because the compost windrow sections of the current study were relatively small and constructed of wood shavings and piglet carcasses without the addition of other carbon and nitrogen sources, microbial activity or temperatures comparable to what would be expected for on-farm mortality compost piles were not able to be sustained. Even under less than ideal composting conditions, PEDV appears to be sufficiently susceptible to composting conditions and comparable to other tested viruses.

The time-temperature trial was designed to examine the effect of temperature on virus persistence in cell culture medium, an optimum environment to test virus stability. Initially, this also included re-isolation of the virus following treatments. PEDV was completely inactivated at the lowest temperature treatment, 37 °C, by 24 h; therefore virus isolation following higher temperature treatments was not performed. Comparable experiments showed cell culture PEDV in virus media was completely inactivated when exposed to ≥60 °C for 30 min [20]. Infectivity was reduced to 0.05% of the original value when heated to 50 °C for 3 h, indicating rapid inactivation at high temperatures [20]. At 37 °C, PEDV retained infectivity after incubation for 6 h, but only at pH 6 to 8 [20]. Although pH was not monitored over the duration of the current trial, the initial pH of virus media was about 7.75, which was within the stable pH range. The upper 95% confidence level of the time required to inactivate 8 log_10_ TCID50 PEDV/mL matrix for minimal essential media, pH 7.2, and temperature 40 °C was 21.7 h [24]. The presence of plasma and alkalinization of the sample potentiated thermal inactivation. Therefore, the inactivation of PEDV under the conditions tested is comparable to other results.

Persistence of PEDV RNA has been tested in many matrices, such as feed, plasma, and manure. Consistently, low temperatures promote virus survival and long-term PCR detection of PEDV RNA, while high temperatures rapidly degrade PEDV. This may explain in part, the seasonal increase in PEDV cases that has been observed during the winter months in the United States following introduction [25]. Additionally, the persistence of PEDV RNA has been reported in open earthen manure storages for up to 9 months where environmental temperatures tend to be lower (−30 to 23 °C) [26]. In controlled laboratory studies, PEDV-spiked manure slurry stored at 25, 4, and −20 °C and PEDV-spiked feed slurry or dry feed maintained at room temperature (about 25 °C) contained RNA for at least 28 days [27]. PEDV-spiked spray-dried bovine plasma contained detectable RNA for at least 21 days at a temperature of 4 °C, although the virus survived less 7 days 22 °C [28]. PEDV-spiked fresh feces exposed to 50 and 60 °C temperatures over a range of relative humidity contained detectable virus RNA up to 7 and 3 days, respectively [27]. The time-temperature trial conducted in this study demonstrated that PEDV RNA persisted in sterile virus media for at least 14 days at temperatures of 37 to 65 °C and was degraded to an undetectable concentration at 70 °C in two of three samples. Compared to those results for PEDV-spiked fresh feces exposed to 50 and 60 °C, this indicates that the complex matrix of manure at high temperatures more effectively degrades RNA. From 37 to 60 °C, the average Cq of media at 14 days was about 30; not a significant difference despite clearly different treatment. RNA was often relatively stable in these treatments after an initial decrease in the first time points. While temperature may provide for virus inactivation and significant RNA decrease in a sterile environment, microbial or enzymatic factors are thought to promote elimination from manure and compost. This provides data to support field observations that while many environmental samples may be positive for PED RNA by qRT-PCR, this detection does not necessarily translate to the presence of infectious virus as RNA is likely detectable for an extended period of time even at elevated temperatures [26].

While this study evaluated outcomes with qRT-PCR, there are limitations of testing for viral RNA. The presence of PEDV RNA does not indicate the presence of infectious virus, which could be detected by cell culture isolation or bioassay techniques. The application of viability PCR has also shown promise in distinguishing infectious and noninfectious viruses in matrices such as water and swine manure, although PEDV has not been tested with this method and studies of virus detection in complex environmental samples, such as manure, are few [29, 30]. However, this may provide a potential method to distinguish this in future studies. In the current study, it is presumed the absence of viral RNA indicates complete destruction of the virus by composting. Extracting virus from compost and PCR techniques both have a limit of sensitivity. The lowest infectious dose of PEDV in feed has been estimated at 5.6 × 10^1^ TCID50/g, well below the detectable limit of virus in this study [31]. While data regarding PEDV persistence in various matrices exposed to high temperature and microbial activity comparable to composting indicates it is readily inactivated, further confirmation could be provided by bioassay in piglets with composted materials.

## Conclusions

Composting appears to be an effective disposal method for PEDV-infected piglet carcasses under the conditions examined. The combination of time and high temperature representative of the compost cycle has been shown to effectively degrade viral RNA in cell culture media, a matrix that promotes stability. Windrow sections achieved a benchmark mean temperature of 55 °C for 4 h at the core during compost cycle 1, which was successful at degrading PEDV RNA. Standard benchmark temperatures for composting also include 40 °C for 120 h and 55 °C for 4 h at all points within the windrow sections during compost cycle 2, which are above the maximum temperatures achieved in this study experimentally. Because these experimental compost windrow sections were constructed of fresh wood shavings and piglet carcasses without the addition of other carbon and nitrogen sources that can improve pile conditions to promote microbial activity, and because these windrow sections were considerably smaller than field-scale compost windrows or piles, temperatures in on-farm animal carcass compost piles commonly achieve and sustain target temperatures much more readily. The complex compost material matrices from experimental compost windrow sections did not contain detectable PEDV RNA by qRT-PCR after one 36-d compost cycle, which supports the premise that greater temperatures sustained for longer periods during on-farm composting will provide effective biosecure disposal of PEDV-infected carcasses. Given our findings of both time and temperature incubations and experimental scale composting, these support the use of on farm composting for virus mitigation on farms.

## Methods

### Time-temperature trial

#### PEDV propagation and quantification

Vero cells were maintained in minimal essential media (MEM) containing 10% fetal bovine serum (FBS) and 100 μg/mL gentamicin. Two-day-old confluent monolayers of Vero cells in 150 cm^2^ flasks were washed two times with MEM containing 2 μg/mL L-(tosylamido-2-phenyl) ethyl chloromethyl ketone (TPCK)-treated trypsin prior to inoculation. Monolayers were infected at approximately 0.01 multiplicity of infection (MOI) of PEDV (USA/Colorado/2013, GenBank accession no. KF272920) in MEM containing 2 μg/mL TPCK-treated trypsin, and incubated at 37 °C until maximum cytopathic effect (CPE) (48 to 96 h). Flasks were cycled through two brief freeze-thaw cycles and stored at −80 °C until further processing. For purification, frozen flasks were thawed and the contents centrifuged at 2000 x g for 10 min. The media supernatant was collected, pooled, mixed, and divided into aliquots that were stored at −80 °C until needed.

The PEDV virus material was assessed for the quantity of infectious virus particles. The sample was diluted in MEM TPCK-treated trypsin at 1:10 (10^−1^) and ten-fold serially diluted to 10^−8^. Prepared two-day-old confluent monolayers of Vero cells in 96-well plates were washed two times with MEM TPCK-treated trypsin. For each diluted sample, 50 μL was added to eight wells. The plates were incubated for 72 h at 37 °C and then fixed with 50% methanol-50% acetone for 10 min. The plates were stained with PEDV monoclonal antibody SD6 ascites (Medgene, Brookings, SD, U.S.) for 30 min at 37 °C, washed with PBS 2×, stained with anti-mouse IgG-FITC (Sigma-Aldrich Inc., St. Louis, MO, U.S.), and washed 2× to allow for visualization of infected cells. Stock virus TCID50/mL was determined by using standard Reed and Muench method [32] and was diluted to the elected TCID50/mL for each respective experiment.

#### Application of treatments

The trial was constructed with combinations of 37, 45, 50, 55, 60, 65, 70 °C and exposure times of 0, 1, 2, 3, 4, 5, 7, and 14 days, with three vials (replicates) of virus sample for each time-temperature treatment. Each virus sample was allocated into a sealed 1.5 mL sterile conical vial (Midsci, St. Louis, MO, U.S.) at a volume of 1 mL and was comprised of 1 × 10^5^ TCID50 of cell-culture propagated PEDV in MEM containing 2 μg/mL TPCK-treated trypsin, 10% FBS, and 100 μg/mL gentamicin. The temperature treatment was performed in an incubator (Heratherm General Protocol Incubator, Thermo Fisher Scientific, Waltham, MA, U.S.) and monitored for temperature consistency throughout the trial. Following treatment, the vials were transferred to −80 °C for storage until qRT-PCR.

For times 0 and 1 day, virus isolation was completed using 500 μL of the treated sample. The virus sample was concentrated by centrifugation at 100,000 x g for 1 h at 4 °C (Beckman Coulter Optima L-90 K Ultracentrifuge, Brea, CA, U.S.) and the pellet re-suspended in 500 μL of MEM containing 2 μg/mL TPCK-treated trypsin. For each sample, 50 μL was added to eight wells of a 96-well plate containing 2-day-old Vero cells which had been washed two times with MEM TPCK-treated trypsin. After 3 days of incubation at 37 °C, the cells were fixed and indirect fluorescence performed with PEDV monoclonal antibody as previously described to classify as PEDV positive or negative.

#### Extraction of RNA

Time-temperature trial samples diluted 1:2 (to conserve sample) and rectal swabs eluted into MEM containing 100 μg/mL gentamicin were subjected to qRT-PCR analysis. For viral RNA extraction, 250 μL of each sample was aliquoted into a tube and extracted using a viral RNA isolation kit (TRIzol, Invitrogen, Carlsbad, CA, U.S.) according to the manufacturer’s instructions, with the addition of 2 μL RNase-free glycogen to the aqueous phase. At least one negative extraction control consisting of all reagents and normal horse serum was included in each extraction. RNA pellets were reconstituted in 20 μL RNase-free water and stored at −20 °C until PCR.

#### Quantitative real time reverse-transcription PCR

Reagents for the PEDV qRT-PCR assay were obtained from a commercially available reaction kit (QIAGEN One Step RT-PCR reagent kit; QIAGEN, Valencia, CA, U.S.), with the addition of magnesium (Sigma-Aldrich Inc., St. Louis, MO, U.S.). The PEDV primers and probes were developed according to a previously published method [33] and ordered from a commercial supplier (IDT, Iowa City, IA, U.S.). The RT-PCR reaction mix consisted of 5 μL 5× Reaction Buffer, 1 μL nucleotide triphosphates, 2 μL of 25 μM/mL MgCl_2_, 5 μL nuclease-free water, 2 μL PEDV forward and reverse primers (10 μM each), 1 μL PEDV HEX-labeled probe (5 μM), 1 μL One Step RT-PCR Enzyme mix, and 8 μL extracted RNA. Each RT-PCR sample was analyzed on a Cepheid Smart Cycler Detection System (Cepheid, Sunnyvale, CA, U.S.) under the following conditions: 50 °C for 30 min; 95 °C for 15 min; and 45 cycles of 94 °C for 30 s, 60 °C for 60 s with optics on, and 72 °C for 30 s. Validated PCR positive controls consisting of PEDV RNA and negative extraction controls were included in each run. Samples were considered positive if the mean fluorescence exceeded 30 fluorescent units prior to 40 cycles and negative and positive PCR controls were properly classified.

#### PCR quantification

**Fig. 4:**
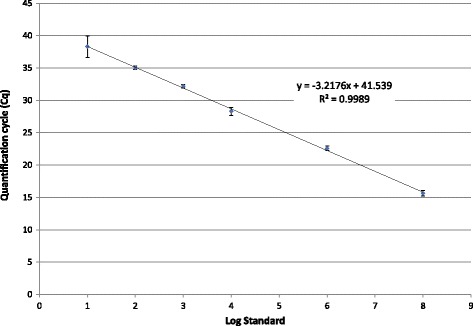
Log of PEDV standard nucleic acid target (8.82 to 8.82E + 8 copies/reaction). Error bars represent standard deviation

**Fig. 5:**
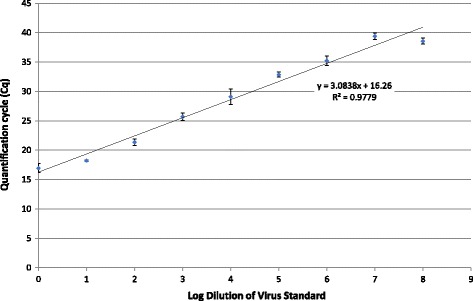
Log dilution of cell culture propagated PEDV (1.00E + 6 to 1.00E-3 TCID50/mL). Error bars represent standard deviation

The amplified PEDV RT-PCR cDNA product was cloned using a commercial kit according to the manufacturer’s directions using 4 μL of PCR product and the provided vector (TA Cloning Kit with One Shot TOP10 Chemically Competent *E. coli*, pCR™4-TOPO® Vector; Invitrogen, Carlsbad, CA, U.S.). Transformed colonies were selected on lysogeny broth agar plates containing 100 μg/mL ampicillin. The plasmid DNA was purified using a commercial kit (PureLink Quick Plasmid Miniprep Kit; Invitrogen, Carlsbad, CA, U.S.), and DNA was quantified with ultraviolet spectrophotometry (SmartSpec 3000 Spectrophotometer, Bio Rad Laboratories, Hercules, CA, U.S.). Copy number was calculated based on the double-stranded DNA plasmid size of 4154 bp. The nucleic acid concentration equivalent to 8.82 × 10^12^ copies was utilized for serial dilutions and subsequent qRT-PCR analysis (Fig. 4). Serial dilution of PEDV virus was performed to compare quantification cycle (Cq) values to TCID50/mL equivalents (Fig. 5).

### Compost trial

#### Windrow composting bin construction

**Fig. 6:**
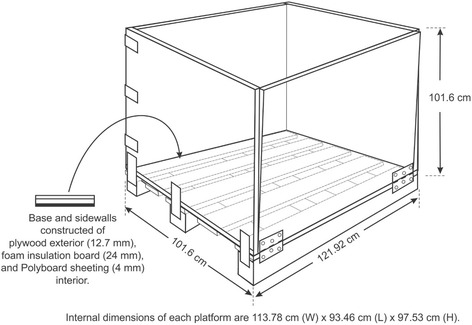
Windrow compost bin design

Insulated platforms were constructed on wood pallets measuring 121.92 cm (W) × 101.6 cm (L). Internal dimensions of platforms were 121.92 cm (W) × 93.46 (L) cm × 97.53 cm (H) to contain a windrow section (Fig. 6). Platform walls were constructed of an outer layer of plywood (12.7 mm) and an inner layer of non-porous PolyBoard sheeting (4 mm). Foam board insulation (24 mm) was placed between these layers to achieve a composite R-value of 17.3 m^2^KW^−1^. This effort was taken to simulate the linear continuation of the windrow and the insulative properties of a compacted soil base.

#### Animal infection

This experiment was conducted in a biosecure (ABSL-2) room at the University of Nebraska – Lincoln Life Science Annex. All procedures involving animals were in accordance with the UNL Institutional Animal Care and Use Committee and Institutional Biosafety Committee. Twenty-seven, 21-day-old piglets were acquired from a PEDV-naïve herd and tested negative for PEDV by qRT-PCR via rectal swab on arrival to the facility. The sows from the herd of origin were serologically negative by PEDV indirect immunofluorescence assay (IFA). The piglets were allowed 3 days of acclimation prior to the start of the study and maintained on a commercial diet free of porcine-origin ingredients.

Virus stock and MEM were mixed so that each piglet inoculum was a 5-mL volume containing 1 X 10^6^ TCID50 of PEDV. This inoculum was administered to piglets via syringe and gavage needle immediately following dilution.

#### Piglet testing and necropsy

Fecal swabs were collected at 3 and 5 days post-infection to confirm infection via viral shedding. Rectal swabs eluted into minimal essential media (MEM) containing 100 μg/mL gentamicin were subjected to qRT-PCR analysis.

Humane euthanasia and necropsy were performed on day 5 post-infection. Fresh and formalin fixed tissues were collected, including two segments of jejunum, one segment of ileum, and mesenteric lymph node. The remaining carcass was incorporated into the compost bin housed within the biosecure room.

#### Compost windrow section construction

Windrow test sections were constructed in an environmentally controlled, biosecurity level 2 room (4.27 m × 3.66 m) at the University of Nebraska-Lincoln Life Sciences Annex, Lincoln, NE. A single trial was conducted with three windrow test sections. Room conditions were maintained at 16–27 °C and 50–80% relative humidity for the duration of the trial.

**Fig. 7:**
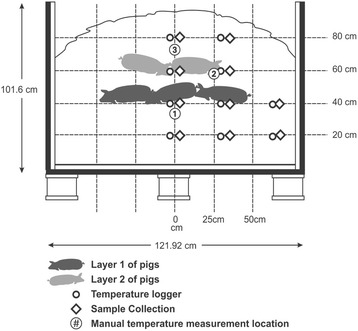
Windrow section construction, temperature monitoring locations, and compost sampling locations

Pine wood shavings (Tractor Supply Co., Lincoln, NE, U.S.) were placed in the bottom of each compost bin to a depth of 20 cm and water from a municipal source was added to wet the material to a target moisture content of 55–60% w.b. Construction of each windrow section was accomplished in 20-cm layers to accommodate placement of temperature loggers. Water addition was performed following each layer of construction. Grab samples of carbon material were collected throughout windrow section construction for analysis of actual moisture content. Once a windrow section depth of 40-cm was achieved, 10 cm of shavings were placed in the pile and three animals were placed along the centerline of the windrow section with minimal space between animals (Fig. 7). A 10-cm layer of wood shavings was placed across the entire windrow section and temperature loggers were placed on the surface of this layer. Two additional animals were then placed along the centerline of the windrow section directly over the temperature loggers. At least 30 cm of clearance was maintained between the animals and platform walls for both layers of animals. The second layer of animals was covered with 20 cm of wetted wood shavings followed by an additional 20 cm of dry shavings to cap the sections with a slight mounding shape along the centerline of the windrow.

#### Compost pile temperature monitoring

Temperature in the windrow sections was measured on a 25-cm × 20-cm spatial grid (Fig. 7) with Apresys Temp Trak Temperature Recorders (Apresys Inc., Duluth, GA, U.S.). Symmetry was assumed along the vertical centerline of the windrow section to minimize the number of spatial sampling locations. Temperatures were recorded on a temporal sampling interval of 20 min throughout each compost cycle. Manual temperature measurements were also collected throughout each compost cycle at three locations in each windrow section (Fig. 7) using a long-handled compost thermometer (REOTEMP Instrument Corp., San Diego, CA, U.S.). The thermometer was inserted and allowed to stabilize for at least 1 min prior to temperatures being recorded.

#### Windrow management

Two consecutive compost cycles, lasting 36 and 37 d, respectively, were performed. Manual temperature monitoring was conducted regularly (daily during the first week of each compost cycle and at least every 3 days beyond the first week of each cycle) to monitor progression of the compost process. When temperatures failed to increase, or declined, water was added to windrow sections from a municipal source to achieve a damp sponge feel of the carbon material. In response to a slow temperature rise in all windrow sections, 5.5 kg of Roebic compost accelerator (Roebic Laboratories Inc., New Haven, CT, U.S.) was added to each windrow section on day 2 of the first compost cycle. On day 5, 0.4 kg of granular fertilizer (Lesco 18–0-18 fertilizer, Home Depot, Lincoln, NE, U.S.) was added to all windrows to further accelerate microbial activity. To add both the compost accelerator and granular fertilizer, carcasses were carefully uncovered and the products were evenly distributed across the exposed surface. Carbon material was then returned to the original configuration following fertilizer addition. Once manual temperature measurements reflected a sustained temperature of less than 100 °C in all windrows, windrows were disassembled, temperature recorders were retrieved, compost material samples were collected, and windrows were reconstructed following the same protocol described previously to initiate a second compost cycle. During the second compost cycle, temperatures were monitored as previously described and moisture added as needed. As with the first compost cycle, once temperature measurements reflected a sustained temperature of less than 100 °C in all windrows, windrows were disassembled for retrieval of temperature recorders and collection of compost material samples and remaining materials were incinerated.

#### Compost sample collection and processing

Compost samples were collected at ten locations in each windrow section corresponding to temperature recorder locations (Fig. 7) at the completion of the first and second compost cycles. To collect samples, windrow sections were carefully deconstructed in layers until temperature recorders were exposed. Grab samples of approximately 50 g of compost material consisting of wood and carbon material with or without decomposed tissue as available per sample site were then collected near the location of each temperature recorder and placed into sterile Whirl-pak bags (Nasco, Fort Atkinson, WI, U.S.). A new pair of nitrile exam gloves was worn during each individual sample collection. Samples were stored at −80 °C until testing.

For processing, 20 g of sample was placed into a sterile Whirl-pak bag along with 50 mL of MEM containing 100 μg/mL gentamicin. The bag was closed and subjected to stomacher blending (Stomacher® 400 Circulator; Seward Limited, Worthing, West Sussex, UK) for 2 min at 230 rpm, assuring that all compost material and organic matter had been thoroughly washed with media solution. The supernatant media was separated into a 50-mL conical vial and stored at −80 °C until qRT-PCR. This procedure was demonstrated to reliably detect 3 × 10^4^ TCID50/ g of compost using the same compost material spiked with a known amount of the previously generated cell culture PEDV.

#### Necropsy tissue analysis

The formalin fixed tissues were routinely processed and embedded in paraffin blocks. The tissues were sectioned at 4 μm, stained with hematoxylin and eosin, and examined with light microscopy. Immunohistochemistry was performed on the same formalin-fixed paraffin-embedded tissues examined histologically. One section was evaluated for each tissue. The sections were cut at 4 μm and applied to slides, which were deparaffinized and stained using an automated immunohistochemical stainer (BenchMark ULTRA; Ventana Medical Systems, Inc., Tucson, AZ, U.S.). The primary antibody consisted of anti-PEDV monoclonal mouse ascites (Medgene, Brookings, SD, U.S.). Positive and negative controls consisted of a slide containing known positive tissue, which was used previously for validating this IHC procedure, along with slides of test samples using an irrelevant antibody: negative mouse serum. After deparaffinization on the immunohistochemistry stainer, the slides were incubated with a cell conditioning solution for 64 min. Primary incubation was for 1 hr at 40 °C. Secondary antibody incubation and staining were conducted with commercial reagents using manufacturer recommended protocols. Tissues were counterstained with hematoxylin for 4 min and coverslipped with glass coverslips. The slides were examined with light microscopy for positive immunoreactivity.

### Data analysis

Data analysis was performed using version 9.4 of SAS software package (SAS Institute Inc., Cary, NC, U.S.).

## Abbreviations

AEV: avian encephalomyelitis virus
AI: avian influenza
Cq: quantification cycle
FMD: foot-and-mouth disease
NDV: Newcastle disease virus
PEDV: porcine epidemic diarrhea virus
qRT-PCR: quantitative real-time reverse transcription polymerase chain reaction
RRT-PCR: real-time reverse transcriptase polymerase chain reaction
TCID50: 50% tissue culture infective dose
USDA: United States Department of Agriculture
